# Effects of Sodium Reduction on the Physicochemical Characteristics of Pretzel Bites

**DOI:** 10.1111/1750-3841.71077

**Published:** 2026-05-06

**Authors:** Reichell P. Cruz Cabrera, Pablo Torres Aguilar, Oguz Kaan Ozturk, Naiman Khan, Florin Dolcos, Damir D. Torrico

**Affiliations:** ^1^ Department of Food Science and Human Nutrition University of Illinois Urbana‐Champaign Urbana Illinois USA; ^2^ College of Applied Health Sciences University of Illinois Urbana‐Champaign Urbana Illinois USA; ^3^ Department of Psychology, Neuroscience Program, and Beckman Institute University of Illinois Urbana‐Champaign Urbana Illinois USA

**Keywords:** physicochemical properties, pretzel bites, quality, sodium reduction, texture

## Abstract

Excessive sodium intake is a global health concern that is largely driven by processed foods. This study evaluated the effects of sodium chloride (NaCl) reduction on the physicochemical properties of pretzel bites. Samples were formulated with five NaCl levels (T1 [0% reduction], T2 [15.91% reduction], T3 [29.28% reduction], T4 [40.53% reduction], T5 [50% reduction]) and assessed for moisture, color, water activity (*a_w_*), salinity, texture profile analysis (TPA), porosity (image‐based), dough volume, and pH. Findings revealed that dough moisture was significantly affected by salt concentration, though final product (baked) moisture remained unchanged. Moderate salt reductions resulted in lighter crust and crumb colors. *a_w_* showed a significant decrease at 50% NaCl reduction, suggesting a water‐binding disruption at this level. Salinity decreased proportionally to reductions in NaCl concentration. Textural parameters, including hardness and chewiness, increased with greater salt reduction, while springiness remained stable. Porosity was highest at moderate NaCl reduction levels and decreased under further reductions. During fermentation, lower NaCl concentrations resulted in increased dough volume and more rapid acidification, as evidenced by lower pH values. Overall, salt reduction affected multiple quality parameters of pretzel bites. Moderate reductions may support healthier formulations while preserving physical functionality. These findings highlight the importance of understanding salt reduction's effects on the quality of baked foods.

**Practical Applications:**

Results showed that moderate sodium reductions produced minimal changes compared to the control (no reduction), suggesting that partial reformulation can maintain product quality while improving nutritional profiles. These findings provide a practical framework for reducing sodium in other baked products by identifying tolerance thresholds in product development, thereby supporting industry efforts to meet public health sodium‐reduction targets without compromising functional quality.

**Abbreviations**
a*: Red–green intensity (color parameter)
*a_w_*
: Water activity
b*: Yellow–blue intensity (color parameter)
CV (%): Coefficient of variation
L*: Lightness (color parameter)
NaCl: Sodium chloride
*P*r > *F*
: Probability value from ANOVA indicating statistical significance
T1: Pretzel bites formulation with 0% sodium chloride reduction (control)
T2: Pretzel bites formulation with 15.91% sodium chloride reduction
T3: Pretzel bites formulation with 29.28% sodium chloride reduction
T4: Pretzel bites formulation with 40.53% sodium chloride reduction
T5: Pretzel bites formulation with 50% sodium chloride reduction
TPA: Texture profile analysis

## 1 Introduction

Excessive sodium intake has been a significant public health concern worldwide due to its strong connections with various chronic diseases. Numerous studies have linked high sodium intake to serious health risks, including cerebrovascular disease, heart disease, ventricular hypertrophy, kidney damage, and other organ impairments (Strazzullo et al. 2012; He and MacGregor 2009; Aburto et al. 2013; Mozaffarian et al. 2014; Nurmilah et al. 2022). One of the most well‐documented consequences of excessive sodium intake is its direct contribution to hypertension, a major risk factor for cardiovascular diseases. The urgency of addressing sodium overconsumption is underscored by the fact that nearly half of the US population and over 1.13 billion people globally suffer from hypertension (Mills et al. 2020). Moreover, the lifetime risk of developing hypertension in Americans is estimated to be as high as 90% (Chobanian et al. 2003). In developed countries, most sodium consumption derives from processed and packaged foods rather than the discretionary usage of table salt (sodium chloride), highlighting the critical need to reformulate food products for public health as consumers become increasingly health‐conscious (Mattes and Donnelly 1991; Brown et al. 2009). Key contributors to sodium consumption include sandwiches (21%), as well as rice, pasta, and grain‐based mixed dishes (U.S. Department of Agriculture and U.S. Department of Health and Human Services 2020).

Numerous studies have investigated sodium reduction strategies in bakery products, finding that moderate NaCl reductions (20%–30%) can be achieved without compromising quality when formulation and processing conditions are optimized. Codină et al. (2021) reviewed the critical multifunctional role of salt in enhancing dough rheology, loaf volume, color, texture, and sensory attributes, and noted that targeted adjustments (e.g., hydration, mixing time, fermentation control, salt replacers) can maintain product integrity in reduced‐sodium formulations.

One of the most popular snack foods that heavily relies on salt for its characteristic taste and texture is the pretzel bites. With a market that exceeded $1.2 billion in the United States, pretzel bites are widely consumed as a low‐fat alternative to fried snacks (Seetharaman 2014). The production of pretzel bites involves several key processing steps, including dough mixing, shaping, alkali cooking, and a two‐step baking process, where sodium is incorporated at multiple stages either as sodium chloride (NaCl) for taste and texture enhancement or sodium hydroxide (NaOH) during the alkali treatment for surface browning and crust formation (Yao et al. 2007). Given their increasing popularity, pretzel bites contribute substantially to daily sodium intake, raising concerns about their potential impact on health, particularly regarding hypertension and cardiovascular diseases.

Recent consumer‐focused studies show that it is possible to gradually reduce the salinity in bread without negatively affecting product acceptance. These studies typically start with a standard formulation containing 2% salt and apply gradual and successive reductions of approximately 10%–14% of that original value. Antúnez et al. (2016) conducted a sequential paired‐comparison test (*n* = 303) and found that such incremental reductions did not significantly impact overall liking. These findings show that gradual sodium reduction strategies can be a feasible solution in staple products such as pretzel bites, suggesting that progressive, modest reductions in salt are more effective than abrupt cuts for maintaining consumer satisfaction (Antúnez et al. 2016).

Moreover, salt plays a fundamental role in yeast‐leavened products such as bread and pretzel bites, affecting taste, texture, fermentation, and overall product quality (Cauvain 2015). Although it comprises only 1%–2% of the total weight, salt significantly affects gluten structure, dough rheology, and yeast activity (Simsek and Martinez 2016). Reducing or omitting salt weakens dough structure, impairs expansion, and diminishes textural attributes, lowering consumer acceptability (Avramenko et al. 2018).

Technological and public‐health initiatives favor a “stealth salt reduction” approach, where sodium in processed foods is gradually reduced without consumer awareness. For instance, the United Kingdom achieved a 25% total sodium reduction in products such as bread and soups, through successive 10% annual cuts (Eyles et al. 2013). Applying this approach to pretzel bites, with stepwise reductions at each production stage (e.g., in dough preparation and post‐bake salting), could reduce sodium content significantly while preserving taste, texture, and appearance.

Although salt reduction has been explored in various bakery products, a significant gap remains in research specifically addressing its effects on fermented dough systems, and particularly, very few studies have focused on pretzel bites, where fermentation dynamics and gas retention are critical to the final product quality (Codină et al. 2021). It is hypothesized that moderate sodium reductions will preserve or enhance certain desirable quality attributes, while excessive reductions may compromise fermentation performance, structural integrity, and overall product quality. This research aims to investigate the effects of sodium reduction in pretzel bites by examining their physical and chemical properties.

## 2 Materials and Methods

### 2.1 Preparation of Pretzel Bite Samples

**FIGURE 1:**
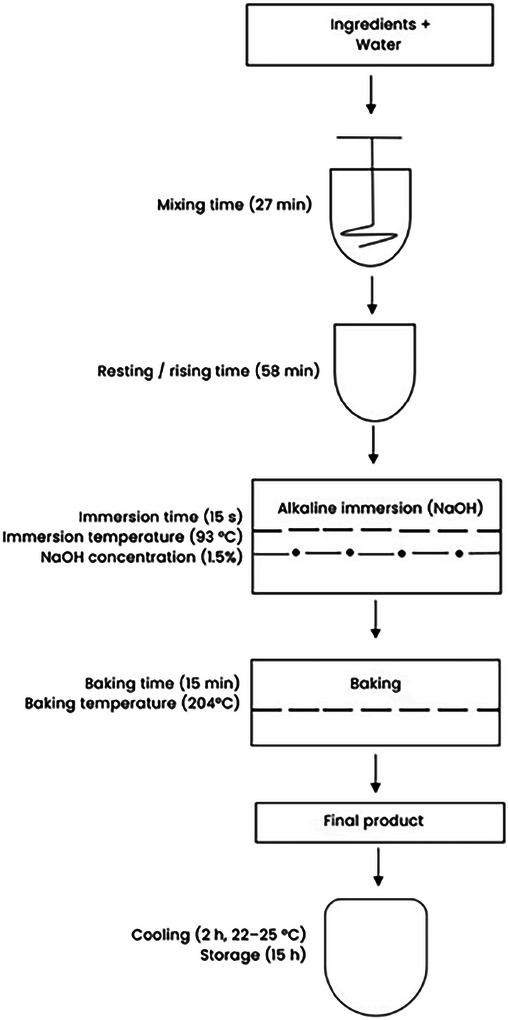
Process flowchart for pretzel bites production.

The pretzel bites preparation method was adapted from Seetharaman (2014), with specific modifications (Figure 1). Ingredients included nonfat dry milk (Carnation Instant, Nestlé, Vevey, Switzerland), all‐purpose enriched flour, light brown sugar, unsalted sweet cream butter (Walmart Inc., Bentonville, Arkansas, USA), active dry yeast (Fleischmann's, AB Mauri Food Inc., Chesterfield, Missouri, USA), and table salt (Great Value, Walmart Inc., Bentonville, Arkansas, USA).

Pretzel bite manufacture involved dough preparation, pretzel shaping through a low‐pressure extruder, immersion in heated alkaline solution, and baking. Proper gluten development and overall dough functionality depended on water incorporation and mixing duration (Groff 2001). Dough was prepared using a bread maker (CBK‐200, Cuisinart, Stamford, Connecticut, USA) under the “Dough/Pizza Dough” setting (Cycle No. 10), which includes 27 min of kneading (3 min using Knead 1 and 24 min using Knead 2), and 58 min of rising (the unit remained inactive, allowing dough to rest, which is critical for flavor and texture development). The temperature of the dough was kept at ∼32°C by dissolving 50 g of nonfat dry milk powder in 200 mL of water heated to 38°C before mixing, to ensure uniform extrusion and improved structural integrity (Seetharaman 2014).

After soft extrusion, pretzel bites (30 ± 2 g each) were immersed in a 1.5% NaOH solution (to improve textural characteristics) at 93°C for an immersion time of 15 s (Aly and Hafez 2020). The modified formulation proposed by Abdi et al. (2024) was implemented, incorporating five levels of progressive NaCl reduction (−15.91%, −29.28%, −40.53%, and −50%), along with a control sample that followed the original formulation (Table 1). These levels were selected based on preliminary trials and focus group sessions aimed at identifying the thresholds of acceptability and technological feasibility for salt reduction in pretzel bites formulations.

**TABLE 1:** Formulations of pretzel bite samples.

Ingredients	T1 (0% reduction)	T2 (15.91% reduction)	T3 (29.28% reduction)	T4 (40.53% reduction)	T5 (50% reduction)
Flour	100.00 g	100.96 g	101.76 g	102.43 g	103.00 g
Yeast	2.00 g	2.00 g	2.00 g	2.00 g	2.00 g
Salt	6.00 g	5.05 g	4.24 g	3.57 g	3.00 g
Sugar	5.00 g	5.00 g	5.00 g	5.00 g	5.00 g
Nonfat dry milk	80.00 g	80.00 g	80.00 g	80.00 g	80.00 g
Butter	10.00 g	10.00 g	10.00 g	10.00 g	10.00 g
Salt level (% of original)	100 (control)	84.09	70.72	59.47	50

After baking at 204°C for 15 min, pretzel bites were allowed to cool at room temperature (22°C–25°C) for 2 h on baking trays. Once cooled, samples were stored in labeled Ziploc bags at room temperature until further analysis. Physicochemical measurements were conducted after 15 h of storage to ensure consistent sample handling across treatments.

### 2.2 Physicochemical Measurements of Pretzel Bites

#### 2.2.1 pH and Volume of the Dough

The pH and volume of the dough were evaluated to monitor the fermentation dynamics. pH measurements were performed using a digital food pH meter (Model YY‐1030 V, Yewhick, Shenzhen, China). Samples of 100 g of dough from each treatment were analyzed at two different time points (0 and 1 h) at a controlled temperature of 23°C (Groff 2001).

For the volume assessment, 250 mL glass beakers were used, with each initially filled with dough up to the 50 mL mark. The beakers were covered with plastic film to prevent moisture loss and incubated at a controlled temperature of 23°C (Groff 2001). The increase in dough volume was measured after 1 h of incubation time to evaluate the extent of fermentation‐driven expansion.

#### 2.2.2 Moisture Content

To measure moisture content, 5 g of both pretzel bites and dough samples from each treatment were used. The analysis was carried out using a moisture analyzer (USS‐HMA01, U.S. Solid, Cleveland, Ohio, USA). Standard drying mode was employed, which means that the instrument heated the sample to a preset temperature (120°C), and maintained that temperature steadily until the drying process was completed. This ensures consistent and reproducible moisture determination across all samples.

#### 2.2.3 Color Measurements

**FIGURE 2:**
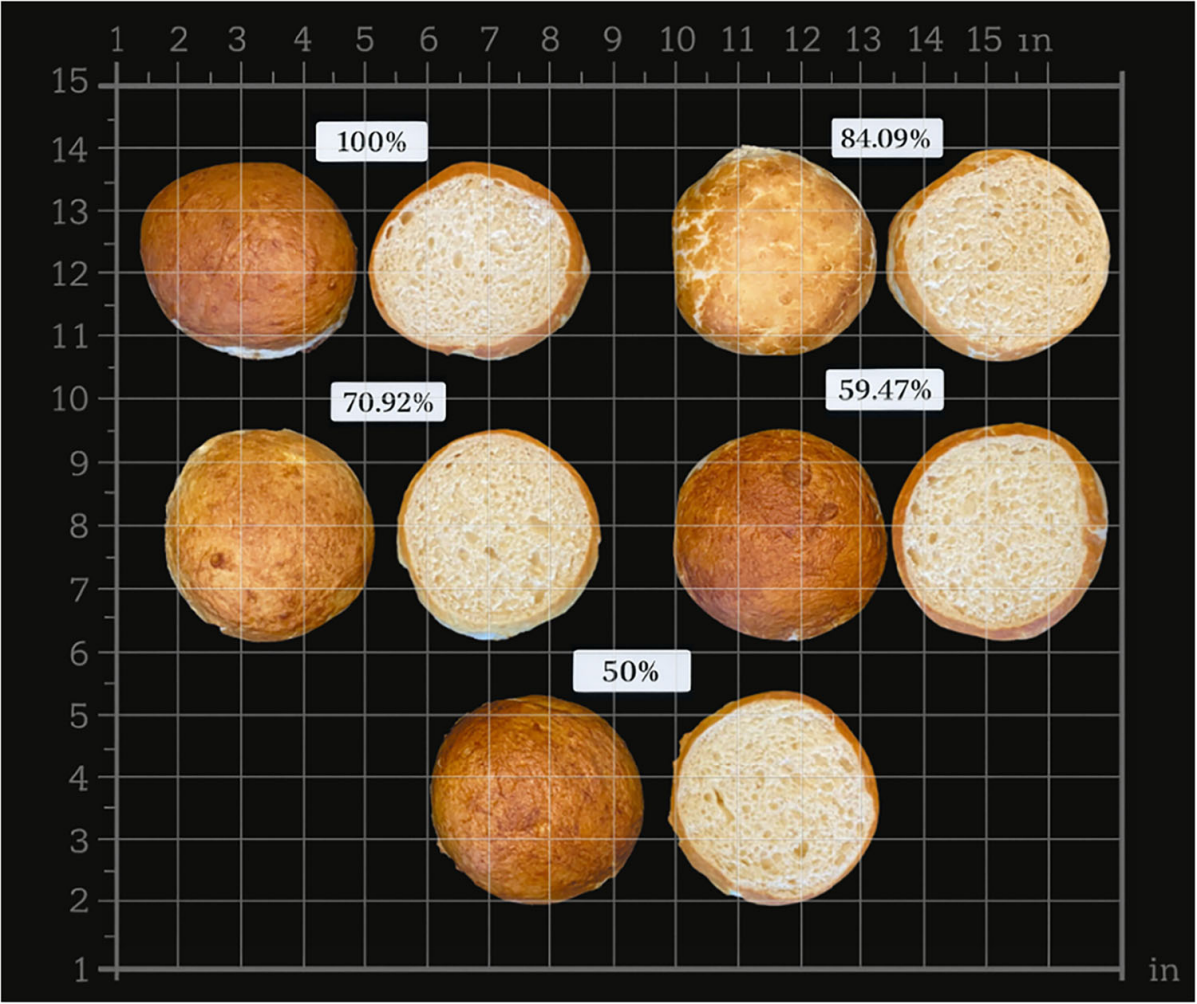
Crusts and crumbs sides of the pretzel bites.

Color analysis was performed on both the crumb and crust of the pretzel bites. A colorimeter (WR‐10, DahoMeter, Dongguan, China) equipped with an 8 mm measurement aperture and a D65 light source evaluated color parameters, including L* (lightness), a* (red–green intensity), and b* (yellow–blue intensity). Color was measured at different points on the crust and crumb surfaces to capture representative variability (Figure 2).

#### 2.2.4 Water Activity (*a_w_*)

*a_w_* of the dough and the pretzel bites was measured using an *a_w_* meter (HD‐4B, NADE Instruments, Hangzhou, China). The instrument operated within a working temperature range of −10°C to 50°C and a relative humidity (RH) range of 0%–95% RH. The instrument's accuracy was ±0.3°C for temperature and ±0.015 *a_w_* for *a_w_* at 23°C ± 5°C.

#### 2.2.5 Texture Profile Analysis (TPA)

TPA was conducted on pretzel bites using a texture analyzer (TA.XTplus100, Stable Micro Systems, Surrey, UK) with the Texture Expert software (Version 1.0, Stable Micro Systems), following the method proposed by Gåmbaro et al. (2002) and Kurek et al. (2019). For the analysis, a cylindrical aluminum compression probe with a 25 mm diameter (P/25) was used. Pretzel bites cut into cubes measuring 20 × 20 × 20 mm were subjected to a two‐bite compression protocol to generate a texture profile curve (Kadan et al. 2001), using the following settings: pretest speed of 2.0 mm/s, test speed of 3.0 mm/s, posttest speed of 3.0 mm/s, 40% deformation, relaxation time of 5 s, a trigger force of 10 g, and using the auto mode feature for the trigger type. This method allows for a more accurate representation of the product's texture during chewing compared to single‐compression or low‐deformation texture tests.

Hardness was determined as the maximum force (Peak Force 1, expressed in Newtons) required to compress the sample during the first compression cycle. Springiness was calculated as the ratio of the distance (or time) between the start and end of the second compression to that of the first compression, reflecting the ability of the sample to recover its shape after deformation. Cohesiveness was obtained from the ratio of the area under the second compression curve to the area under the first, indicating the structural integrity of the sample during deformation, and is therefore a dimensionless parameter with values lower than 1. Finally, chewiness was computed as the product of hardness, springiness, and cohesiveness, representing the force required to chew the sample (Gåmbaro et al. 2002).

Stickiness was defined as the maximum negative force (expressed in Newtons, N) recorded during probe withdrawal from the sample, reflecting the adhesive characteristics of the pretzel bite surface.

#### 2.2.6 Salinity

The salinity of the pretzel bites was measured using a conductivity‐based salinity meter (PAL‐SALT, Cat. No. 4250, Hanna Instruments, Woonsocket, Rhode Island, USA). The device operates using the conductivity method, with an accuracy of ±0.05% for salt concentrations ranging from 0.00% to 0.99%, and a relative precision of ±0.5% for concentrations between 1% and 10%. To prepare the sample, 50 mL of distilled water was mixed with 10 g of ground pretzel bite sample, which had been processed using a hand blender (Handheld Immersion Blender, Proctor Silex, Glen Allen, Virginia, USA). The mixture was stirred for 10 min at 550 rpm using a magnetic stirrer (LACHOI, Guangzhou, China). The salinity percentage was calculated by multiplying the measured salt concentration by a dilution factor of 16.67%.

#### 2.2.7 Air Pockets (Porosity by Image Analysis)

The presence and distribution of air pockets (porosity) in the pretzel bite samples were analyzed using an image analysis procedure developed by Dr. Torrico (customized Python‐based tool, University of Illinois Urbana‐Champaign). High‐resolution images of the pretzel bites were captured using a smartphone camera (iPhone 11, Apple Inc., Cupertino, California, USA) in portrait mode.

**FIGURE 3:**
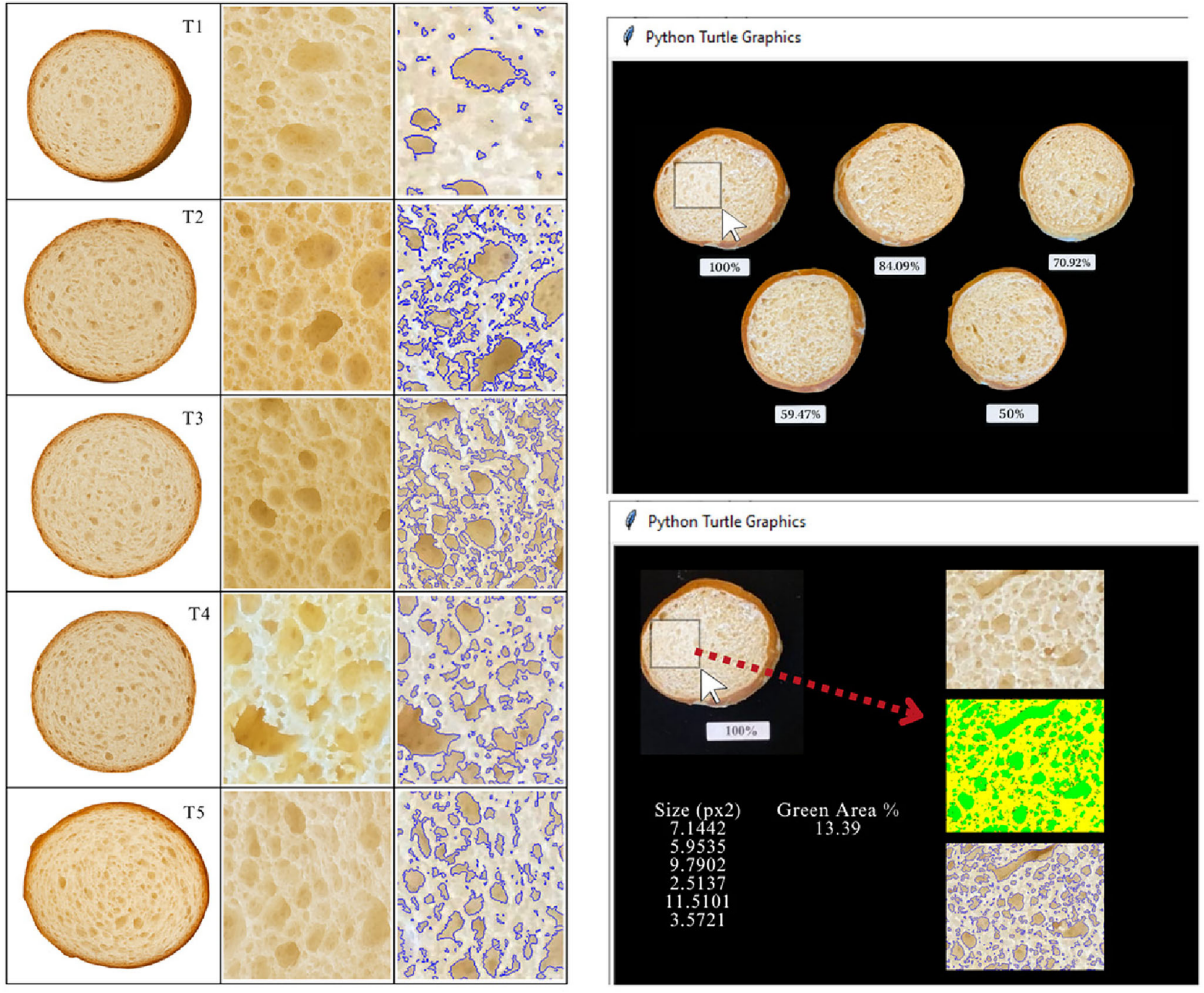
Air pocket of the pretzel bites.

Using the image analysis tool, air pockets were manually segmented within a selected region of each pretzel bite sample. Air pocket size was initially quantified as pixel area and subsequently converted to mm^2^ through image calibration, while air pocket area (%) was calculated as the proportion of the analyzed region occupied by air pockets relative to the total area. For each treatment, 15 pretzel bite samples were analyzed, with one representative region selected per sample to quantify porosity. Measurements at the individual pore level were subsequently averaged to generate representative values for each treatment (Figure 3).

#### 2.2.8 Experimental Design and Statistical Analysis

Three independent batches of pretzel bite dough were prepared on different days, representing three experimental replications. For each batch and treatment level, five replicates were analyzed for all physicochemical parameters, including moisture content, *a_w_*, salinity, pH, volume, color, texture, and air pocket distribution. The statistical analysis was conducted using SAS software (SAS Institute Inc., Cary, North Carolina, USA). A one‐way analysis of variance (ANOVA) was performed to determine significant differences between treatment means. Tukey's honest significant difference (HSD) test was used as a post‐hoc comparison method when significant differences were detected, with a confidence level set at *p <* 0.05. Principal component analysis (PCA) was performed using the XLSTAT software (Addinsoft, Paris, France) to visualize the relationship between samples and identify patterns across the salt‐reduced pretzel bite formulations.

## 3 Results and Discussion

### 3.1 pH and Volume of the Dough

Table 2 shows the dough volume and pH value at 0 and 1 h of fermentation across the five treatments (T1–T5). Statistical comparisons were conducted within each fermentation time point.

**TABLE 2:** pH and volume of the dough (pretzel bites).

Treatment	Time (hours)	pH value	Volume (mL)
	Mean ± standard deviation		
T1 (control)	0 h	5.57 ± 0.08^a^	50 ± 0^a^
T2		5.65 ± 0.11^a^	50 ± 0^a^
T3		5.73 ± 0.13^a^	50 ± 0^a^
T4		5.69 ± 0.04^a^	50 ± 0^a^
T5		5.75 ± 0.07^a^	50 ± 0^a^
T1	1 h	5.61 ± 0.07^b^	70 ± 26^b^
T2		5.56 ± 0.13^a^	100 ± 20^b^
T3		5.62 ± 0.06^ab^	107 ± 11^b^
T4		5.60 ± 0.04^b^	183 ± 29^a^
T5		5.58 ± 0.07^b^	167 ± 29^a^
CV (%)		12.17	1.32
*P*r > *F*		< 0.01	< 0.01

*Note*: Means with different letters within the same fermentation time are significantly different (*p* < 0.05). *P*r > *F* = ANOVA probability. If *P*r > *F* < 0.05, there are significant differences between treatments. T1 (0% salt reduction), T2 (15.91% salt reduction), T3 (29.28% salt reduction), T4 (40.53% salt reduction), and T5 (50% salt reduction).

Abbreviation: CV = coefficient of variation.

At 0 h, no significant differences in dough volume were observed among treatments, as all samples remained at the initial volume (50 mL). Likewise, pH values at this time point did not differ significantly among treatments, indicating that salt reduction had no immediate effect on the initial dough conditions before fermentation began.

After 1 h of fermentation, significant differences in dough volume were observed. Treatments T4 and T5 exhibited the highest volumes, followed by T3 and T2, while the control (T1) showed the lowest expansion. This indicates that reduced salt levels enhanced yeast activity and gas production during the early fermentation stage. Fermentation occurs as yeast grows and the dough traps the released gases, allowing it to expand under internal pressure. Yeast growth is supported by the presence of sufficient *a_w_*, fermentable sugars, and the activity of amylolytic enzymes that convert starches into maltose, a sugar that yeast can metabolize. Meanwhile, the dough's ability to retain gases is primarily governed by its rheological properties, which dictate its structure and elasticity (Carcea et al. 2020).

These findings align with Lynch et al. (2009), who reported that NaCl plays a regulatory role in dough fermentation, with higher salt levels reducing yeast activity and dough expansion. Consequently, reducing salt levels can enhance yeast activity and increase CO_2_ production.

### 3.2 Moisture of Dough and Pretzel Bites

The results indicate that there were no significant differences (*p* > 0.05) in the moisture content of the baked pretzel bites across the various salt treatments (Table 3). In contrast, small numerical differences were observed in the dough moisture prior to baking; however, ANOVA indicated no significant differences among treatments. Specifically, T3 (29.28% reduction) and T4 (40.53% reduction) exhibited lower dough moisture compared to other treatments. However, this pattern was not consistently maintained in the final baked product. For example, T4 and T5 (40.53% and 50% reduction) displayed slightly higher average moisture (40.29% ± 6.11% and 40.29% ± 3.04%, respectively) than other treatments with higher salinity, and T1 and T5 had similar moisture values despite their large differences in salinity levels. These inconsistencies indicate that, although measurable, the observed differences in dough moisture were small in magnitude, did not follow a consistent trend with salt reduction level, and were not retained after baking. Therefore, they are more likely associated with normal experimental variability rather than a systematic formulation effect related to salt concentration.

**TABLE 3:** Physicochemical parameters of pretzel bite samples.

S	Treatment	Moisture (%)	*a_w_*	Salinity (%)	Air pocket area (%)	Air pocket size (mm^2^)
Dough	T1	38.21 ± 0.74^a^	0.93 ± 0.01^a^	—	—	—
	T2	37.90 ± 2.55^a^	0.94 ± 0.01^a^	—	—	—
	T3	35.04 ± 2.06^b^	0.94 ± 0.01^a^	—	—	—
	T4	35.02 ± 2.42^b^	0.94 ± 0.01^a^	—	—	—
	T5	38.47 ± 1.00^a^	0.94 ± 0.02^a^	—	—	—
CV (%)		0.00	1.10	—	—	—
*P*r > *F*		0.01	0.04	—	—	—
Pretzel bites	T1	38.29 ± 2.15^a^	0.95 ± 0.01^a^	0.85 ± 0.02^a^	17.55 ± 2.86^d^	22.49 ± 49.35^a^
	T2	38.06 ± 2.80^a^	0.95 ± 0.00^a^	0.76 ± 0.05^b^	35.26 ± 3.85^b^	17.81 ± 54.89^a^
	T3	38.57 ± 1.41^a^	0.94 ± 0.01^a^	0.71 ± 0.04^c^	41.86 ± 3.07^a^	32.59 ± 58.94^a^
	T4	40.29 ± 6.11^a^	0.94 ± 0.01^a^	0.69 ± 0.03^c^	11.15 ± 1.23^e^	8.17 ± 17.87^a^
	T5	40.29 ± 3.04^a^	0.93 ± 0.00^b^	0.69 ± 0.05^c^	31.23 ± 3.69^c^	24.26 ± 53.06^a^
CV (%)		9.28	0.66	5.48	11.48	229.18
*P*r > *F*		0.25	< 0.01	< 0.01	< 0.01	0.41

*Note*: Means with different letters within the same column are significantly different (*p* < 0.05). *P*r > *F* = ANOVA probability. If *P*r > *F* < 0.05, there are significant differences between treatments. T1 (0% salt reduction), T2 (15.91% salt reduction), T3 (29.28% salt reduction), T4 (40.53% salt reduction), and T5 (50% salt reduction).

Abbreviation: CV = coefficient of variation.

Although salt concentration is known to influence dough water absorption and gluten network development (Isaak et al. 2019; McCann and Day 2013; Preston 1989), the thermal process during baking likely minimized moisture differences by evening out initial variability. Heat application drives off water and modifies structural components (starch gelatinization and protein denaturation), resulting in a more uniform moisture distribution across treatments (Amani et al. 2022). Previous work on bread and pretzel‐type products has shown that baking parameters such as temperature and time often have a stronger effect on the final moisture content than the initial dough composition (Isaak et al. 2019).

Other studies have reported that moderate NaCl reductions (20%–30%) can be implemented without significantly affecting dough hydration, gluten development, or final product texture, if mixing and fermentation conditions are optimized (Codină et al. 2021; Belz et al. 2012). Our data partially align with these observations that, despite measurable differences in dough moisture, the baked pretzel bites’ moisture content remained relatively stable across treatments, with no clear linear relationship to salt concentrations. Similarly, Monteiro et al. (2021) observed that salt reduction in Portuguese bread had a limited effect on crumb moisture, while still affecting other parameters such as salinity and color. This reinforces the idea that in our study, moisture in the baked product was likely governed more by baking‐induced water loss and structural changes than by salt concentration alone.

### 3.3 a_w_

The results obtained for the *a_w_* of both the baked pretzel bites and their corresponding doughs with different salt formulations are shown in Table 3. The reduction in *a_w_* observed in pretzel bite samples with 50% NaCl reduction (T5) may be attributed to structural changes in the gluten network caused by low salt levels, which can alter water distribution and binding within the dough matrix (McCann and Day 2013). NaCl, due to its hygroscopic nature, binds free water molecules in food matrices, thereby decreasing the amount of water available for microbial activity (Hutton 2002). This increase in osmotic pressure leads to cellular dehydration, which inhibits microbial growth. In baked products such as bread, NaCl plays a crucial role in controlling the proliferation of spoilage organisms, including mold and *Bacillus* species, ultimately contributing to an extended shelf life (Silow et al. 2016).

Interestingly, a nonlinear trend was observed where *a_w_* did not decrease proportionally with salt reductions. Treatments T1 to T4 had comparable *a_w_* values, suggesting that reductions above 50% are needed to observe significant changes in water binding behaviors. The reduction in *a_w_* observed in the pretzel bite samples with 50% NaCl (T5) was significantly different than those of the other treatments, indicating a measurable but limited change in water‐binding behaviors at this level of salt reduction. Changes in pretzel bites matrix interactions during baking can also affect *a_w_*. At low concentrations, salt stabilizes the gluten network by shielding the charged sites on gluten proteins, which reduces electrostatic repulsion and allows the proteins to interact more closely, resulting in a stronger dough structure (Kinsella and Hale 1984). However, at higher concentrations, salt interacts more extensively with water molecules, and its effects on gluten formation become more dependent on specific ions’ activities (McCann and Day 2013). These interactions can affect both water retention and the mobility of molecules within the dough matrix.

### 3.4 Salinity

Table 3 shows the salinity for all baked pretzel bite treatments. The results show a significant decrease in salinity percentages as the concentrations of NaCl in the treatments decreased. The highest salinity was observed in T1 with a value of 0.85% ± 0.02%, which was significantly higher than the other treatments (*p <* 0.05). As the NaCl concentration decreased (T2–T5), salinity also decreased, reaching a plateau at T4 and T5, with both registering a salinity of 0.69% ± 0.03% and 0.69% ± 0.05%, respectively, with no significant difference between them (Table 3). This trend can be attributed directly to the dilution of NaCl, a major contributor to the total salinity in aqueous systems. Since NaCl is a strong electrolyte and dissociates fully in solution (Timko et al. 2010), reducing its concentration naturally leads to a proportional decrease in measured salinity. This confirms that added NaCl was the dominant contributor to salinity in this context, as is consistent with findings in similar studies involving controlled salt treatments (Torrico et al. 2019). Despite a 9.47% decrease in NaCl concentration from T3 to T4, no significant change in salinity was observed, suggesting a potential threshold effect. This could be due to the buffering capacity of the medium or saturation limits in the ion retention and conductivity (Koksal et al. 2016).

### 3.5 Air Pockets (Porosity)

The analysis of air pocket areas and sizes in baked pretzel bites revealed significant differences among treatments with varying NaCl concentrations (Table 3). These internal structures are key indicators of porosity in baked goods and are directly related to texture, sponginess, and sensory acceptance (Demirkesen and Ozkaya 2018). Regarding the air pocket area percentage, 70.72% NaCl (T3) exhibited the highest proportion (41.86% ± 3.07%), followed by 84.09% NaCl (T2) with a similarly high value (35.26% ± 3.85%). Both treatments’ air pocket values were significantly higher than those of other treatments (*p* < 0.05), suggesting that moderate salt reductions favor a more open and airy crumb structure. This outcome aligns with findings made by Belz et al. (2012) and Yovchev et al. (2017), who reported that intermediate salinity (1.0%–1.5%, weight basis) improve gluten elasticity and gas retention capacity in wheat bread by strengthening the gluten network, whereas very low salt levels weaken the structure, and excessive salt concentrations make the structure overly rigid, ultimately reducing loaf volume. Similarly, Beck et al. (2012) observed that salt modulates gluten tenacity and extensibility, thereby influencing gas retention.

T4 resulted in the lowest air pocket area and the smallest air pocket size among all treatments, indicating the formation of a more compact crumb structure. These factors can promote the development of fewer but larger gas cells, partially compensating for any weakening of the gluten network. This nonlinear effect of salt on dough and bread structure has been reported in previous studies (Beck et al. 2012; Belz et al. 2012), in which the relationship between salinity and air pocket size was not strictly linear. While intermediate salt levels (T2–T3) clearly favored larger and more abundant air pockets, high salt (T1) and very low salt (T5) levels still produced moderate values, suggesting that multiple factors, such as dough viscosity, yeast activity, and baking expansion, interact with salinity to determine the final porosity. For air pocket sizes, T3 exhibited the largest average value (32.59 ± 58.94 mm^2^), followed by T1 (22.49 ± 49.35 mm^2^). T4 had the smallest pocket sizes (8.17 ± 17.87 mm^2^), reinforcing the idea that adequate, but not excessive, salinity is crucial for optimal crumb development (Carcea et al. 2020) (Figure 3).

The large dispersion observed for air pocket size is inherent to the image‐based porosity analysis, which captures individual pore dimensions across multiple regions of the pretzel crumb. This approach reveals pronounced structural heterogeneity, where a small number of large air pockets markedly increases variability, resulting in high standard deviation and coefficient of variation (CV) values.

Overall, the results demonstrate that NaCl reduction affected multiple physicochemical parameters in the pretzel bites, although the magnitude and nature of these effects varied across variables. While moisture content in the final product was not significantly affected, dough moisture showed clear sensitivity to salt levels. This suggests that NaCl plays a more prominent role in water absorption during dough preparation than in the retention of moisture after baking. As previous studies have shown, this may be due to salt's capacity to tighten the gluten structure, reducing the water‐binding sites available during mixing (Isaak et al. 2019; Preston 1989).

### 3.6 Color

The color characteristics of pretzel bites’ crumbs and crusts were notably affected by NaCl reductions, with statistically significant differences observed across treatments (Table 4). Salt plays a critical role in dough structure formation by modulating gluten development, gas retention, and fermentation rates (Conforti and Davis 2006). Changes in these structural properties can affect crumb lightness by altering gas cell size and distribution, which affects light scattering. Moderate salt reduction (e.g., T2) enhanced yeast activity and gas cell expansion, resulting in a lighter crumb (higher L* value) compared to the control (T1), in agreement with Pashaei et al. (2022) and Beikzadeh et al. (2017).

**TABLE 4:** Color analysis of pretzel bites.

Form	Treatment	L*	a*	b*
		Mean ± standard deviation		
Crumb	T1	51.52 ± 4.02^b^	−0.22 ± 0.14^b^	14.03 ± 0.40^bc^
	T2	55.09 ± 1.26^a^	−0.41 ± 0.08^a^	14.39 ± 0.46^c^
	T3	52.40 ± 2.96^ab^	−0.26 ± 0.13^b^	13.85 ± 0.54^b^
	T4	52.15 ± 3.84^b^	−0.26 ± 0.13^b^	13.93 ± 0.59^bc^
	T5	51.65 ± 0.61^b^	−0.24 ± 0.08^b^	15.42 ± 0.80^a^
	CV (%)	5.04	−39.64	3.45
	*P*r > *F*	< 0.01	< 0.01	< 0.01
Crust	T1	35.51 ± 4.07^d^	13.18 ± 0.60^b^	30.70 ± 2.45^c^
	T2	45.27 ± 2.25^b^	12.47 ± 0.70^b^	34.85 ± 2.19^ab^
	T3	51.21 ± 1.69^a^	11.20 ± 0.85^c^	36.43 ± 1.04^a^
	T4	40.68 ± 4.78^c^	13.99 ± 0.56^a^	31.91 ± 4.42^bc^
	T5	40.75 ± 2.05^c^	13.95 ± 0.46^a^	30.23 ± 5.04^c^
	CV (%)	7.40	5.33	10.32
	*P*r > *F*	< 0.01	< 0.01	< 0.01

*Note*: Means with different letters within the same column are significantly different (*p <* 0.05). *P*r > *F* = ANOVA probability. If *P*r > *F* < 0.05, there are significant differences between treatments. T1 (0% salt reduction), T2 (15.91% salt reduction), T3 (29.28% salt reduction), T4 (40.53% salt reduction), and T5 (50% salt reduction).

Abbreviation: CV = coefficient of variation.

However, the trend was not strictly linear; samples with greater salt reduction (T3–T5) did not consistently show higher L* values. This suggests that excessive salt reduction may trigger different mechanisms, such as over‐fermentation, collapse of gas cells, or changes in moisture retention, that counteract lightness gains observed at moderate reduction levels (Sun et al. 2020).

In the crust, the control (T1) exhibited the darkest surface (lowest L* value), while reduced‐salt treatments generally produced lighter and more yellowish crusts (higher b* values), particularly T3 (70.72% NaCl). This aligns with the literature that shows that sodium interacts with the Maillard browning, affecting the surface pH, *a_w_*, and sugar availability of baked products (Pasqualone et al. 2019; Moreau et al. 2009). Nevertheless, T5 (50% NaCl), in our present study, did not have the highest L* or b* values, indicating that beyond a certain reduction threshold, other factors may affect color, such as pH and sugar–amino acid interactions (Belz et al. 2012; El Hosry et al. 2025), resulting in a paler pretzel appearance.

Color attributes, both in crust and crumb, were particularly responsive to salt variation. Treatments with lower salt levels generally resulted in lighter colors, likely due to enhanced yeast activity and reduced Maillard browning efficiency. This observation is consistent with the findings made by Pashaei et al. (2022) and Pasqualone et al. (2019), who noted similar color alterations in reduced‐sodium bread products. Such effects are related not only to surface browning mechanisms but also to fermentation dynamics and sugar retention, further illustrating the interconnected nature of these physicochemical parameters.

### 3.7 TPA

The analysis of the pretzel bites revealed significant differences (*p* < 0.05) among the treatments with different NaCl concentrations in the parameters of hardness, cohesiveness, stickiness, and chewiness (Table 5). However, no significant differences were observed in springiness (*p* = 0.23), suggesting that the elasticity of the product was not substantially affected by variations in salt concentrations. Hardness increased progressively as the NaCl content was reduced from 100% to 59.47%, reaching its peak in treatment T4 (6.35 ± 0.08 N). This trend may be attributed to the lower salinity, which can influence gluten network formation and water distribution during thermal processing, ultimately contributing to a firmer texture (Carcea et al. 2020). However, a further reduction from T4 to T5 resulted in decreased hardness, which may be due to an excessive weakening of the gluten network, reducing the dough's ability to retain water and resist deformation, thereby producing a softer texture (McCann and Day 2013; Silow et al. 2016; Lynch et al. 2009). Previous research by Lynch et al. (2009) demonstrated that NaCl enhances the strength of the gluten network in bread doughs by promoting the formation of thicker protein strands. This strengthening effect is thought to occur because salt masks the surface charges of gluten proteins, thereby minimizing electrostatic repulsion and facilitating stronger protein interactions (McCann and Day 2013).

**TABLE 5:** Texture profile analysis of pretzel bites.

Treatment	Hardness (N)	Cohesiveness (‐)	Stickiness (N)	Springiness (‐)	Chewiness (N)
	Mean ± standard deviation				
T1	4.80 ± 0.15^d^	0.53 ± 0.01^c^	0.013 ± 0.004^bc^	1.08 ± 0.04^a^	5.18 ± 0.24^d^
T2	5.22 ± 0.16^c^	0.54 ± 0.01^bc^	0.015 ± 0.003^c^	1.08 ± 0.02^a^	5.67 ± 0.19^c^
T3	5.46 ± 0.08^b^	0.58 ± 0.01^a^	0.010 ± 0.001^b^	1.08 ± 0.01^a^	5.91 ± 0.09^b^
T4	6.35 ± 0.08^a^	0.54 ± 0.01^b^	0.012 ± 0.002^bc^	1.07 ± 0.01^a^	6.79 ± 0.14^a^
T5	5.57 ± 0.13^b^	0.50 ± 0.01^d^	0.006 ± 0.001^a^	1.06 ± 0.02^a^	5.92 ± 0.16^b^
CV (%)	2.24	1.74	22.39	2.07	2.92
*P*r > *F*	< 0.01	< 0.01	< 0.01	0.23	< 0.01

*Note*: Means with different letters within the same column are significantly different (*p <* 0.05). *P*r > *F* = ANOVA probability. If *P*r > *F* < 0.05, there are significant differences between treatments. T1 (0% salt reduction), T2 (15.91% salt reduction), T3 (29.28% salt reduction), T4 (40.53% salt reduction), and T5 (50% salt reduction).

Abbreviation: CV = coefficient of variation.

Regarding cohesiveness, T3 showed the highest value (0.58 ± 0.01), suggesting better capacity for the product to maintain its internal integrity during chewing. In contrast, the treatment with the lowest cohesiveness was T5 (0.50 ± 0.01) which may indicate structural weakness due to the reduced protein–salt interactions (Ferrari et al. 2022). With respect to stickiness, the lowest value was observed in T5 (0.006 ± 0.001 N), while T2 showed the highest stickiness (0.015 ± 0.003 N). Chewiness exhibited an increasing trend from T1 to T4, with the latter treatment having the highest value (6.79 ± 0.14 N). This suggests that a moderate reduction in NaCl can contribute to a firmer and more chewable texture, likely due to the formation of a denser gluten network (Lynch et al. 2009). However, excessive salt reduction, as in T5, did not continue this trend, which may be attributed to the weakening of the gluten network, leading to a softer structure despite its higher moisture content. Overall, the results indicate that an intermediate level of salt reduction (e.g., T3 and T4) can improve certain texture attributes without compromising product quality. These findings are consistent with previous research suggesting that salt not only acts as a flavor enhancer but also serves as a texture modulator in baked products (Ferrari et al. 2022).

Globally, texture and structure‐related traits, including hardness, cohesiveness, and air pocket formation, were affected by NaCl concentrations. Intermediate salt levels, particularly T3, favored improved textural performance, whereas lower salt levels resulted in reduced cohesiveness and weaker structural integrity. Excessive reduction (e.g., T5) led to less desirable features, such as decreased cohesiveness and paler crusts, pointing to the existence of a threshold below which the product quality may be compromised. These findings support the dual role of salt as both a structural and sensory modulator in baked products (Ferrari et al. 2022; Beck et al. 2012; Lynch et al. 2009).

### 3.8 PCA

**FIGURE 4:**
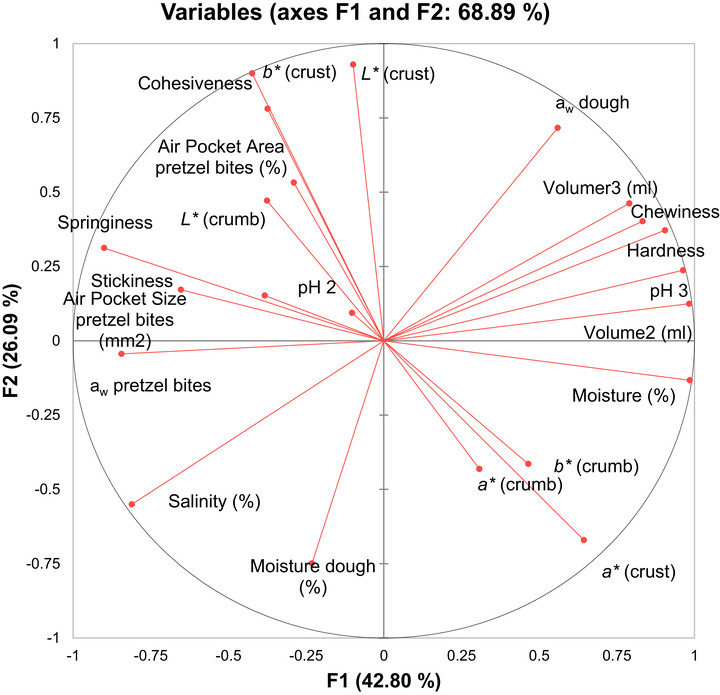
Principal component analysis (PCA) showing the physicochemical attributes of the pretzel bites.

These trends were further supported by PCA, which explained 68.89% of the total variance through its first two dimensions (Figure 4). The PCA biplot revealed clear associations among variables influenced by NaCl reductions. For instance, hardness, chewiness, *a_w_*, and dough volume clustered together on the positive side of F1, suggesting that these attributes were positively associated with higher salt concentrations. Conversely, moisture of the dough, salinity, and springiness were positioned oppositely, indicating inverse associations. Notably, parameters such as cohesiveness, crumb L*, and air pocket area grouped closely in the upper left quadrant, aligning with treatments that achieved a more desirable structural profile. The positioning of moisture content and color attributes (a*, b*) on the right side of the plot further highlights the multifactorial effect of salt on both visual and textural characteristics.

The reduction of NaCl in pretzel bite formulations presents both strengths and weaknesses from a physicochemical standpoint. On the one hand, moderate reductions allowed for the maintenance of desirable structural and textural properties, particularly when salt levels were not drastically lowered. This suggests that partial sodium reduction is feasible without severely compromising product quality. On the other hand, more extreme reductions (e.g., T5) resulted in less favorable outcomes, such as diminished cohesiveness, lighter crust coloration, and altered moisture dynamics in the dough. These findings point to a functional threshold below which NaCl reduction may lead to significant deterioration in key quality attributes. Thus, while sodium reduction remains a valuable strategy for improving nutritional profiles, its implementation requires a balanced approach that considers its physicochemical consequences.

## 4 Conclusion

This study demonstrates that moderate NaCl reduction in pretzel bites is feasible without compromising key quality attributes, provided that reductions remain within technologically tolerable thresholds. The results highlight the central role of salt in structuring the dough matrix and modulating texture and crumb characteristics. Among the reduced‐sodium formulations, treatment T3 (29.28% NaCl reduction) emerged as the best overall compromise, as it maintained favorable dough expansion, porosity, texture, and color attributes without the structural weakening observed at higher reduction levels. From an industrial perspective, these findings support the development of lower‐sodium baked snack products by identifying formulation ranges that balance nutritional improvement with functional performance. Given the increasing demand for healthier food options, optimizing salinity while preserving product quality remains a critical challenge for the food industry. Future research should focus on consumer acceptance, shelf‐life stability, and the use of salt replacers or functional ingredients to further enhance reduced‐sodium formulations.

## Author Contributions

**Reichell P. Cruz Cabrera**: conceptualization, investigation, writing – original draft, methodology, formal analysis, data curation. **Pablo Torres Aguilar**: conceptualization, methodology, writing – review and editing, supervision. **Oguz Kaan Ozturk**: methodology, investigation, funding acquisition, writing – review and editing, supervision. **Naiman Khan**: methodology, investigation, funding acquisition, writing – review and editing, supervision. **Florin Dolcos**: methodology, investigation, funding acquisition, writing – review and editing, supervision. **Damir D. Torrico**: conceptualization, investigation, funding acquisition, writing – original draft, methodology, formal analysis, writing – review and editing, supervision.

## Conflicts of Interest

The authors declare no conflicts of interest.
